# S8-4 Reaching beyond competitive sports. How International Sports Federation can use their main competitions to promote physical activity. A World Athletics pilot project

**DOI:** 10.1093/eurpub/ckad133.041

**Published:** 2023-09-11

**Authors:** Paolo Emilio Adami, Frédéric Garrandes, Stephane Bermon

**Affiliations:** World Athletics; LAHMESS, Université Côte d'Azur; World Athletics; LAHMESS, Université Côte d'Azur; World Athletics; LAHMESS, Université Côte d'Azur

## Abstract

**Purpose:**

International Sports Federations (IF) are global governing bodies for each respective sport and their main duties are to organize international competitions, regulate and promote their sport. Despite their objective of promoting sports participation, their activity mostly focused on competitive/elite sports so far. International competitions not only represent the epitome of each sport but could also be used as an opportunity to improve the health of those living in the hosting city or the staff working for the local organising committee (LOC). The purpose of this pilot project was to measure the benefits of active commuting (AC) and leisure time physical activity (LTPA) of the staff involved in the World Athletics Championships Oregon22 during the two weeks of competition.

**Project description:**

World Athletics partnered with StravaMetro□ to measure AC between official accommodation and competition venues, as well as the staff LTPA, during the Championships. Before the event, World Athletics staff was briefed on the project purposes and given information on AC options available in Eugene (pedestrian and cycling paths, bike rental). Staff members were encouraged to record their AC and LTPA through the Strava App. During the 2 weeks, a total of 18,656 walking/running trips (both AC and LTPA) were recorded in Eugene. This represents a 225.4% increase compared to the same period of the previous year. Furthermore, 2690 cycling commuting trips were recorded, representing a 143% increase.

**Conclusions:**

This pilot project demonstrates that IF can efficiently use their main events to promote AC and LTPA to their staff and the workforce involved. Encouraging AC, improves the health of those active, reduces traffic related air pollution, road traffic, and transportation costs for the LOC. An upscaled approach adopted in the early phase of the event planning, involving spectators would amplify observed results and their health-related benefits. The post analysis by StravaMetro□ of the walking and cycling routes used by staff and spectators can also allow the host city to better define and create protected routes which represent a tangible legacy. These objectives require a strong collaboration between the IF, LOC, city government and the national institutions promoting AC and HEPA.

